# Immunohistochemical analysis of soft tissue response to polyetheretherketone (PEEK) and titanium healing abutments on dental implants: a randomized pilot clinical study

**DOI:** 10.1186/s12903-022-02536-0

**Published:** 2022-11-11

**Authors:** Iva Milinkovic, Ana Djinic Krasavcevic, Sasha Jankovic, Jelena Sopta, Zoran Aleksic

**Affiliations:** 1 Department of Periodontology and Oral Medicine, School of Dental Medicine, University of Belgrade, #4 Dr Subotic St., Belgrade, 11 000 Serbia; 2 Institute of Pathology, Faculty of Medicine, University of Belgrade, #1 Dr Subotic St., Belgrade, 11 000 Serbia

**Keywords:** PEEK, Titanium, healing abutment, inflammation, immunohistochemistry

## Abstract

**Background:**

The data on polyetheretherketone (PEEK) influence on the peri-implant soft tissues in clinical settings are deficient. The aims of this pilot study were to analyze and compare soft tissues’ response to PEEK and titanium (Ti) healing abutments (HA) by means of histological and immunohistochemical analyses.

**Methods:**

A total of 22 implants with PEEK or Ti HA were placed in 11 patients, applying the “split-mouth” study design. Three months later, soft tissue specimens were harvested from 20 implants for histology in order to qualitatively detect the inflammatory cells’ presence, to semi-qualitatively analyze the inflammation intensity and to assess the inflammatory responses type by immunohistochemical analysis using LCA, CD3, CD20 and CD68 antibodies.

**Results:**

Epithelial infiltrate followed by an intensive inflammation in sub-epithelium was observed in 100% around PEEK HA. A number of LCA+ and CD 68+ cells was significantly higher in PEEK comparing to Ti group (*p* = 0.001 and *p* = 0.020, respectively), while CD 20+ and CD3+ counted cells were found in a significantly higher amount in Ti than in PEEK group (*p* = 0.006 and *p* = 0.010, respectively).

**Conclusion:**

PEEK HA seems to evoke the more intense tissue inflammatory response demonstrated predominantly by histocytes’ and plasmacytes’ activation, while Ti HA triggers the inflammatory reaction of lower intensity, dominantly mediated by B-cells.

**Trial registration:**

The study registered at ClinicalTrials.gov (NCT04436939).

## Introduction

Titanium dental implants have been thoroughly analyzed over the past decades and have become the treatment of choice for the replacement of the missing teeth [1]. Although high survival rates have been documented, possible complications remain an issue. In spite of differing literature data on peri-implantitis prevalence (from 11 to 47%), most of the studies confirmed that complications may affect around 10% of implants and 20% of patients after 5 to 10 years of dental implants in function [2–4]. Therefore, the need for peri-implantitis prevention on each level is crucial.

A need for an improvement of both osseointegration and peri-implant soft tissue seal maintenance exist so far [5]. Alongside osseointegration process, the peri-implant soft tissue maturation occurs, resulting in an establishment of a barrier epithelium (mucosal seal) and a connective tissue attachment to the implant surface [6, 7]. Peri-implant soft tissues are fundamental, acting as a biologic seal between bone and the oral cavity and a barrier to the contamination of implant surface by pathogenic flora [8]. The importance of inflammation-free mucosal seal and connective tissue barrier, as well as maintenance of a long-lasting physical and structural boundary between the soft tissue and implant collar or abutment surface have been more recently pointed as critical factors in peri-implant health and stability [9].

In respect to the previously mentioned fact, the selection of the abutment material could play one of the essential roles in the soft tissue seal stability and subsequent marginal bone integrity. Therefore, an abutment material should be less prone to biofilm adhesion, and act preventively in terms of soft tissue inflammation. Commonly, implant abutments are made of titanium (Ti); nonetheless, innovative materials such as ceramics and polymers have been introduced in prosthetic implant dentistry. One of the materials more recently imported in implant dentistry is a polymer polyetheretherketone (PEEK), whose pioneer application was in orthopedic surgery [10], showing a beneficial property in terms of lower Young’s (elastic) modulus (3–4 GPa), close to the human bone [11].

PEEK is a synthetic, tooth colored (white) thermoplastic polymeric material with numerous favorable properties, such as good mechanical characteristics and chemical inertness [12]. However, PEEK is still categorized as bioinert owing to its low integration with surrounding tissues, as opposed to titanium [13, 14]. It has been used in dental implantology and prosthodontics as healing abutment, temporary abutment and for various frameworks [15, 16] .

In an in vitro settings, it has been shown that PEEK should perform equally to commercially pure titanium [17] and that biofilm formation on the surface of PEEK is comparable or lower than on the surface of conventionally applied abutment materials such as zirconia and titanium [5]. Another laboratory findings revealed that peripheral blood mononuclear cells produced significantly more proinflammatory cytokines when exposed to the PEEK surface compared to the Ti-6 aluminum-4 vanadium surface [18]. Furthermore, the authors of the more recent animal study evaluated the soft tissue response to dental implant closure caps made of PEEK or Ti by the occurrence of multinucleated giant cells (MNGCs). Significantly more MNGCs were in contact with PEEK than with Ti closure caps, suggesting a more prominent soft tissue response around PEEK closure caps [19]. However, to the best of our knowledge, the current scientific literature lacks clinical data to confirm these observations. Additionally, the literature information on soft tissue response on the PEEK surface, as well as the influence of PEEK on the peri-implant soft tissue interface in the clinical settings are deficient.

Since PEEK has an increasing popularity as a material used in oral implantology and given the fact the scarce scientific evidence lies behind its interactions with the surrounding tissues, the objectives of this pilot clinical study were to assess and thoroughly analyze the soft tissue profile around PEEK healing abutments on osseointegrated implants. Specifically, the primary aim of present study was to histologically analyze and compare soft tissues’ response to PEEK and Ti material, while the secondary aim was further elaboration of the nature of these tissue reactions by means of immunohistochemical analyses.

## Materials and methods

### Study design

This study was conducted at the Department of Periodontology and Oral medicine of the Belgrade School of Dental Medicine in collaboration with the Institute of Pathology of the Belgrade Faculty of Medicine. The study was approved by the Institutional Ethical Committee (36/28). All the subjects enrolled in this study signed an informed consent and all methods were performed in accordance with the Helsinki declaration of 1964. and subsequent amendments.

This was an observational pilot clinical trial, with a “split-mouth” design. It was conducted as an integral part of the further study still in progress, comprising larger number of patients. The study was registered at ClinicalTrials.gov (NCT04436939), on 18/06/2020.

### Study sample

The subjects included in this study were recruited from the patients of the Department of Periodontology and Oral Medicine, School of Dental Medicine, University of Belgrade. Individuals diagnosed with partial edentulism in posterior maxillary or mandibular arches, with healed sites and missing at least one tooth on each side of the jaw were selected, following clinical and radiological examination.

The data obtained from all patients were registered in case record forms created for the needs of this research. Clinical periodontal parameters such as periodontal probing depth (PD), clinical attachment level (CAL), bleeding on probing (BOP) and plaque index (PI) were recorded for each patient. One single calibrated examiner performed the measurements using a periodontal probe (UNC-15 Hu-Friedy, Chicago, IL) in six points around every tooth. The available bone volume was evaluated, and implant diameter and length were planned according to the CBCT images (Cranex® 3D, Soredex, Tuusula, Finland).

A total of 11 patients requiring implant placement (with bilateral single tooth gaps and/or extended edentulous spaces distally from maxillary or mandibular canines) were enrolled in this study if fulfilling the inclusion criteria:

1. Men and non-pregnant women, age 18 to 70;
2. Systemically healthy patients;
3. An adequate bone quantity allowing for the insertion of 3.8 mm or 4.3-mm diameter implants;
4. At least 2 mm of keratinized mucosa at the experimental sites;
5. No active periodontal disease at the time of inclusion/surgery and proper oral hygiene defined by bleeding on probing scores (BOP) and plaque indices (PI) less than 15%.

### Surgical procedure

A single trained surgeon (Z.A.) performed all implant placement procedures under local anesthesia (2% lidocaine with epinephrine, 1:100000). Mid-crestal incisions extended through the sulci of adjacent teeth were made and full-thickness mucoperiosteal flaps were elevated with caution not to interfere with muco-gingival junction. Surgical implant sites were prepared according to the manufacturer’s instructions using drills of increasing dimeters, with a low-trauma surgical technique under a copious irrigation with sterile physiological saline. Thereafter, two bone level implants were inserted (C-TECH Esthetic line (EL), Italy) up to 1 mm subcrestally, with a single-stage protocol.

Ti dental implants placed in the posterior mandible and maxilla were randomly assigned to receive transmucosal healing by either: PEEK healing abutment – PEEK HA (A, test group) or Ti healing abutment- Ti HA (B, control group). Test and control abutments (C-TECH, San Pietro in Casale (BO), Italy) were provided in width of 4.3 mm and in different heights (2, 3, or 4 mm), selected in relation to gingival height.

**Fig. 1:**
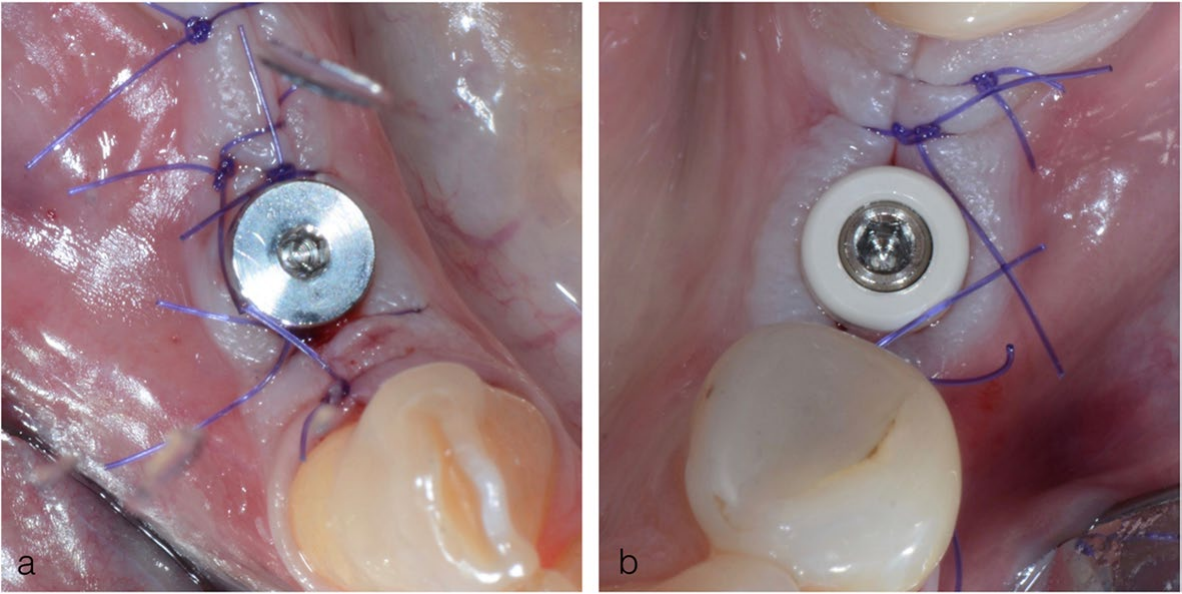
Clinical presentation of PEEK and Ti healing abutments placed after the surgery within the split-mouth protocol

A surgeon (Z.A.), not previously involved in the patient selection, randomly assigned participants following simple randomization procedures to either test or control group. The randomized codes were enclosed in sequentially numbered, identical, sealed envelopes. Following the “split-mouth” study design, implant abutments from randomly chosen group was placed on one jaw side, while the abutment from the remaining group was screwed on the contralateral side of the same jaw (Fig. 1). In the group A, immediately after the implant insertion, a PEEK abutment was screwed, while in the group B, the same procedure was performed using Ti HA. All abutments were connected using a torque of 10 Ncm. Finally, the mucoperiosteal flaps were repositioned and adapted using non-resorbable single sutures to ensure a primary wound closure and transmucosal healing.

### Postoperative care

Patients were prescribed antibiotics (Amoxicillin caps. 500 mg, 3 × 1 or Clyndamicin, 600 mg, 2 × 1, in case of Penicilline allergy) and 0.2% chlorhexidine solution (ASD Curasept 220, Saronno, Lombardia, Italy) during the first post-operative week, as well as non-steroidal anti-inflammatory drugs when needed (Ibuprofen, 600 mg, every 6 hours). Sutures were removed 1 week after the surgery. Patients were thoroughly instructed to perform good oral hygiene daily. No additional periodontal-supportive therapy was performed in a healing period up to the time of tissue specimens harvesting.

### Sampling

Three months following the implant insertion, a control periodontal charting was performed in each patient, including the BOP and PI scores record around inserted implants. Then, a minimally invasive surgical procedure was performed for the placement of larger diameter HA. At this stage, a triangular incision [20] was made at mesial and distal aspect of the existing implant HA, in order to remove both the HA with the band of the surrounding soft tissue of 1–1.5 mm in thickness. In case of reduced tooth-implant space due to single-implant installation, a circumferential incision around HA was performed during tissue harvesting procedure.

Soft tissue specimens were gently rinsed using 4% formalin and fixed in 10% neutral buffered formalin solution. The samples were then processed to the laboratory for further histological preparation.

Finally, commercially available 5 mm wide HA (C-TECH, San Pietro in Casale (BO), Italy) were selected by height according to the site-specific situation and gingival height and applied to ensure a soft tissue conditioning prior to prosthetic restoration.

### Histological preparation and light microscopic analysis

In the laboratory, samples were fixed into 10% neutral buffered formalin for 24 h at room temperature, dehydrated and embedded in paraffin, out of which sections of 4 μm thickness were cut. For histological evaluation, the specimens were stained with hematoxylin-eosin (HE). For inflammatory cells’ quantification, the immunohistochemical stainings stated below were used.

### Histological analysis

All histological analyses were performed at the Institute for Pathology, Faculty of Medicine, University of Belgrade by a single experienced pathologist, blinded to the treatment samples, marked as samples from group A and group B. The analysis was performed in three parts and gradually. Firstly, the aim was to qualitatively detect the presence of inflammatory cells, secondly, to count inflammatory cells and to semi-qualitatively analyze inflammation intensity and finally, to assess the type of the inflammatory responses by immunohistochemical analysis.

The following morphological changes were analyzed: presence, localization and intensity of the inflammation. Presence in epithelial and subepithelial tissues were categorized qualitatively (yes/no) while the intensity was qualified semi-qualitatively (light, medium, and intensive by following criteria: 0–20 positive cells from 50 counted cells “light”, 21–40 positive cells from 50 counted cells “medium” and 41–50 positive cells from 50 counted cells “intensive”.)

### Immunohistochemical analysis

Immunohistochemical techniques were carried out using the avidin-biotin-peroxidase complex method with an LSAB2 kit (Dako, Glostrup, Denmark). The use of avidin-biotin interaction in immuno-enzymatic techniques provides a simple and sensitive method to localize antigens in formalin-fixed tissues [21]. The primary antibodies used in this study were: LCA (M3629Clone 318–6-11, dilution 1:25; Dako, positive control tonsil tissue), CD 3 (M3539 Clone β-catenin1, dilution 1:200; Dako, positive control tonsil tissue), CD 20 (M7240 Clone MIB-1, dilution 1:100; Dako, positive control tonsil tissue), and CD 68 (EPR2241, ab134175, dilution 1:200; Abcam, positive control tonsil tissue). Since CD3 is expressed in all stages of T lymphocytes differentiation, this marker was used to identify both cytotoxic T and T helper cells [22, 23]. As CD20 represents a surface molecule involved in B cells to plasmocytes’ transition, it is frequently used for B-cells recognition [23, 24]. CD68 is expressed by tissue macrophages and histiocytes, therefore it has been used for targeting of these cells [23, 25, 26]. Finally, the use of an antibody to leucocyte common antigen (LCA) labels all leucocyte cell types, since LCA represents a surface glycoprotein expressed on all hematopoietic cells, except on mature erythroid cells [27]. Immunohistochemical positive cells presence on photographs has been counted on a control unit Nikon DS- at 200× magnification in “hot-spots”. Counting of positive and negative images was performed using the ImageJ program with a handmade plug-in. For each antibody was counted 50 cells, in total 200 cells per sample. Cells with cytoplasmic staining for CD68, membrane stained for CD3 and CD20 and both cytoplasmatic and membrane stained for LCA antibodies, were considered as positive in immunohistochemistry.

### Statistical analysis

Data were analyzed using R-Project: R CoreTeam 2021 software (R Foundation for Statistical Computing, Vienna, Austria). In the case of categorical variables, the proportion of cases in each category was compared. For the continuous variables, the differences between the measured values for each material were checked for normality (using the Shapiro-Wilk normality test and by visual inspection using QQ plot) and extreme outliers (based on the boxplot method, i.e. values above Q3 + 3xIQR or below Q1 - 3xIQR), after which the appropriate tests for the comparison of the means of the two groups were applied. If the differences were normally distributed two-tailed paired Student’s t-test was used, whereas two-tailed the Wilcoxon signed-rank test was used if the distribution was non-normal. All *p* values < 0.05 were considered significant.

## Results

### Clinical results

Among 11 patients included in present study, 27% were female and 73% were male, with mean age of 49 years. The patients were predominantly non-smokers (64%). A total of 22 implants were placed in 11 patients. A single patient with two placed implants was excluded from the study due to non-compliance and not recalling to the follow-up examinations. Other 10 patients with 20 placed implants have completed the study period with no adverse events reported. Implant survival and success rate was 100% after 3 months.

The values of examined clinical parameters in 10 enrolled patients (PD, CAL, BOP and PI) prior to implant installation and after 3 months did not demonstrated statistically significant differences between two time points (Two-tailed Wilcoxon signed-rank test; *p* = 0.346, *p* = 0.149, *p* = 0.583, *p* = 0.055, respectively) (Table 1). Furthermore, when observing the BOP and PI around placed implants in the moment of biopsy, significantly greater values were found in PEEK group in comparison to Ti (Two-tailed Wilcoxon signed-rank test; *p* = 0.048 and *p* = 0.010, respectively) (Table 1). However, BOP and PI values around both PEEK and TI were up to 14%, therefore in spite of previously demonstrated statistical significance between groups, the signs of inflammation were not clinically relevant in the time of tissue specimens’ collection.

**Table 1:** Mean and standard deviation of clinical parameters (PD, CAL, PI, BOP) in examined time points

Parameter	Material	n	Mean	SD
PD	baseline	10	2.08	0.123
	3 months	10	2.06	0.107
	difference	10	0.02	0.042
CAL	baseline	10	0.49	0.074
	3 months	10	0.46	0.052
	difference	10	0.03	0.048
BOP	baseline	10	13.4	1.075
	3 months	10	13.2	0.789
	difference	10	0.2	0.632
PI	baseline	10	13.5	0.972
	3 months	10	13.1	0.876
	difference	10	0.4	0.516

### Histological results

This statistical analysis was based on the data from 10 patients. Two materials were compared based on two categorical parameters, infiltrate epithelial (with the values yes/no) and infiltrate subepithelial (with the values light/medium/intensive), and four continuous parameters, LCA, CD68, CD20 and CD3.

**Fig. 2:**
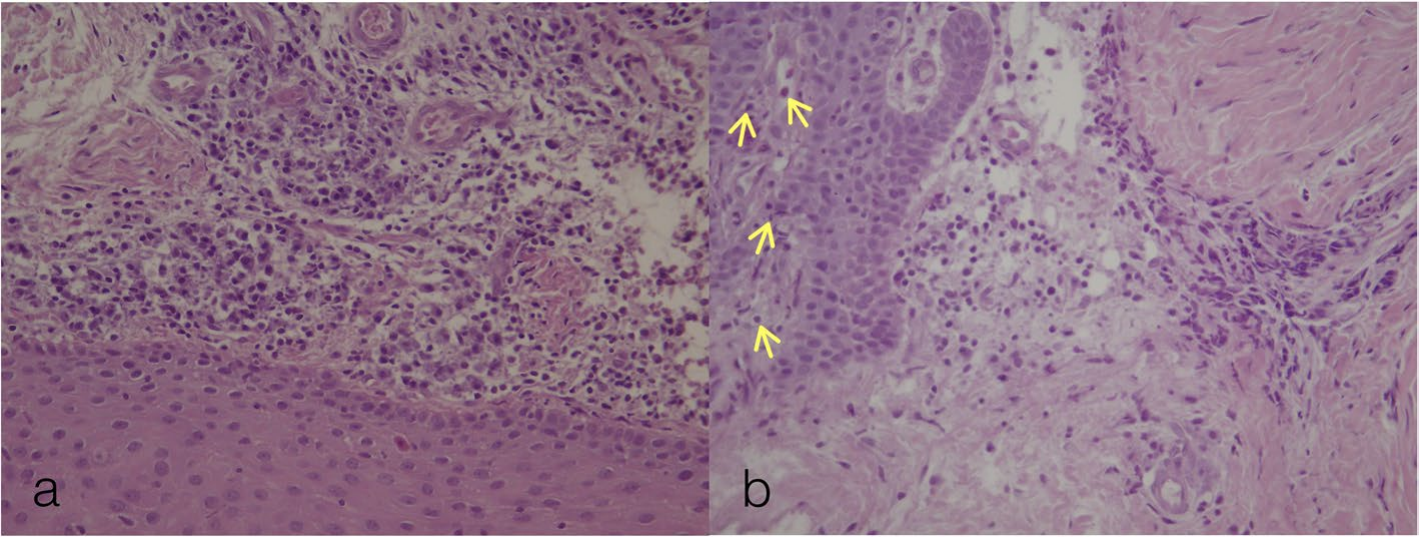
Hematoxylin-eosin staining, elucidating the presence of the inflammatory cells dominantly in subepithelial tissue (3a,3b). Intraepithelial presence of several inflammatory cells was observed in Fig. 3b, as highlighted by the yellow arrows. HE × 400 magnification

**Fig. 3:**
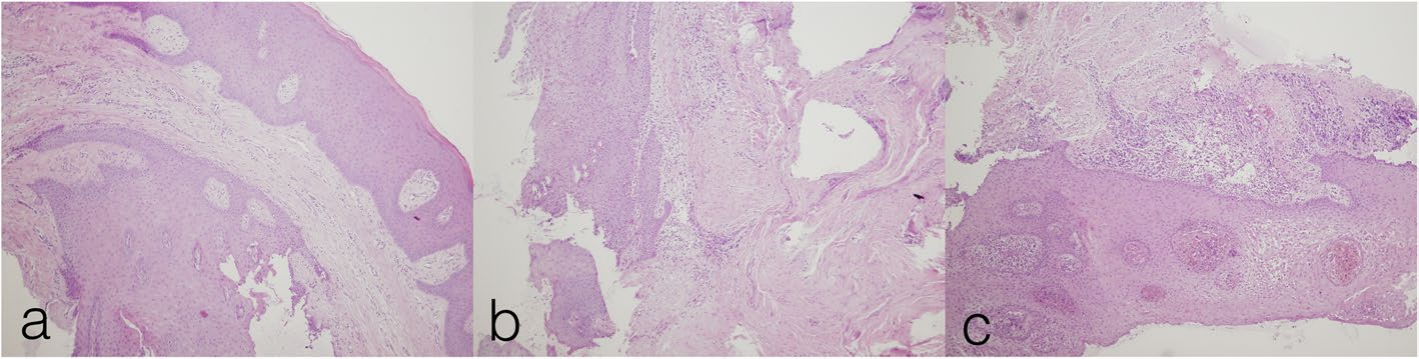
Hematoxylin-eosin (HE) staining of the specimens to assess inflammatory reaction. Inflammatory response was classified as „light” (2a), „medium” (2b) or „intensive” (2c). HE × 100 magnification

In microscopic analysis, on HE stains, following morphological changes were analyzed: presence, localization and intensity of the inflammation. Infiltrate in epithelium was detected in 100% of cases for the material A, while it was not detected for the 80% of cases for the material B (Fig. 2). The null hypothesis that the frequencies are equal for the two materials was rejected (McNemar’s Chi-squared test, *p* = 0.004). Nevertheless, all samples from both groups had inflammatory infiltrate in sub-epithelial tissue. Following the analysis of the distribution of inflammation intensity, infiltrate in sub-epithelium was found to be intensive for 100% of cases in group A, while it was light for 40% of cases and medium for 60% of cases in the group B (Fig. 3). Similarly, the null hypothesis stating the equality of frequencies for the two materials was rejected (McNemar’s Chi-squared test, *p* = 0.046).

**Fig. 4:**
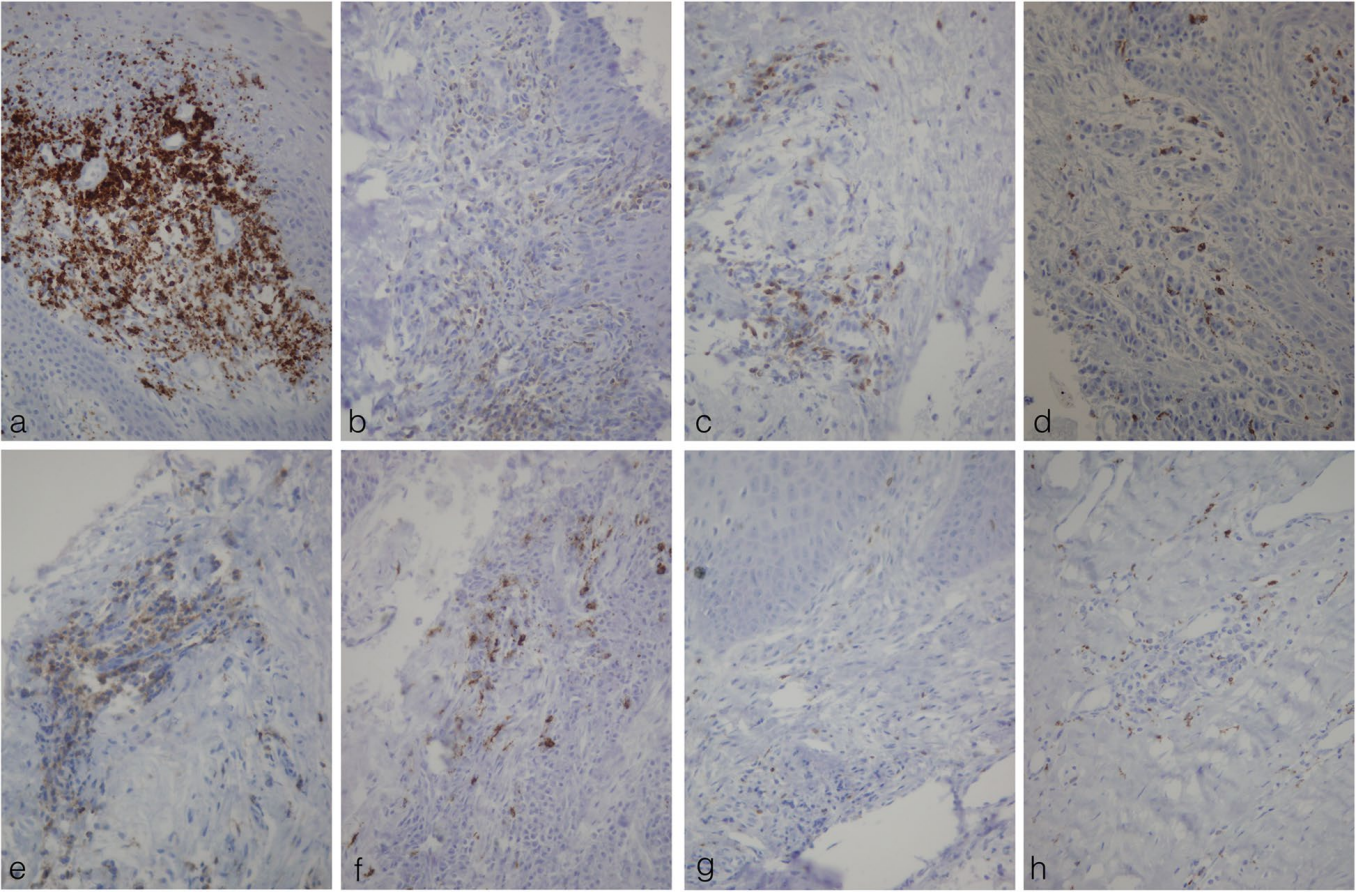
Immunohistochemical analysis of the sections prepared from soft tissues surrounding PEEK and Ti abutments. Comparative presentation of inflammatory cells in specimens appertaining to PEEK group (**a**, **b**, **c**, **d**) and Ti group (**e**, **f**, **g**, **h**) showed respectively more pronounced inflammation around PEEK (**a**, **e**: LCA; **b**, **f**:CD20; **c**, **g**: CD3; **d**, **h**: CD68). ×400 magnification

**Fig. 5:**
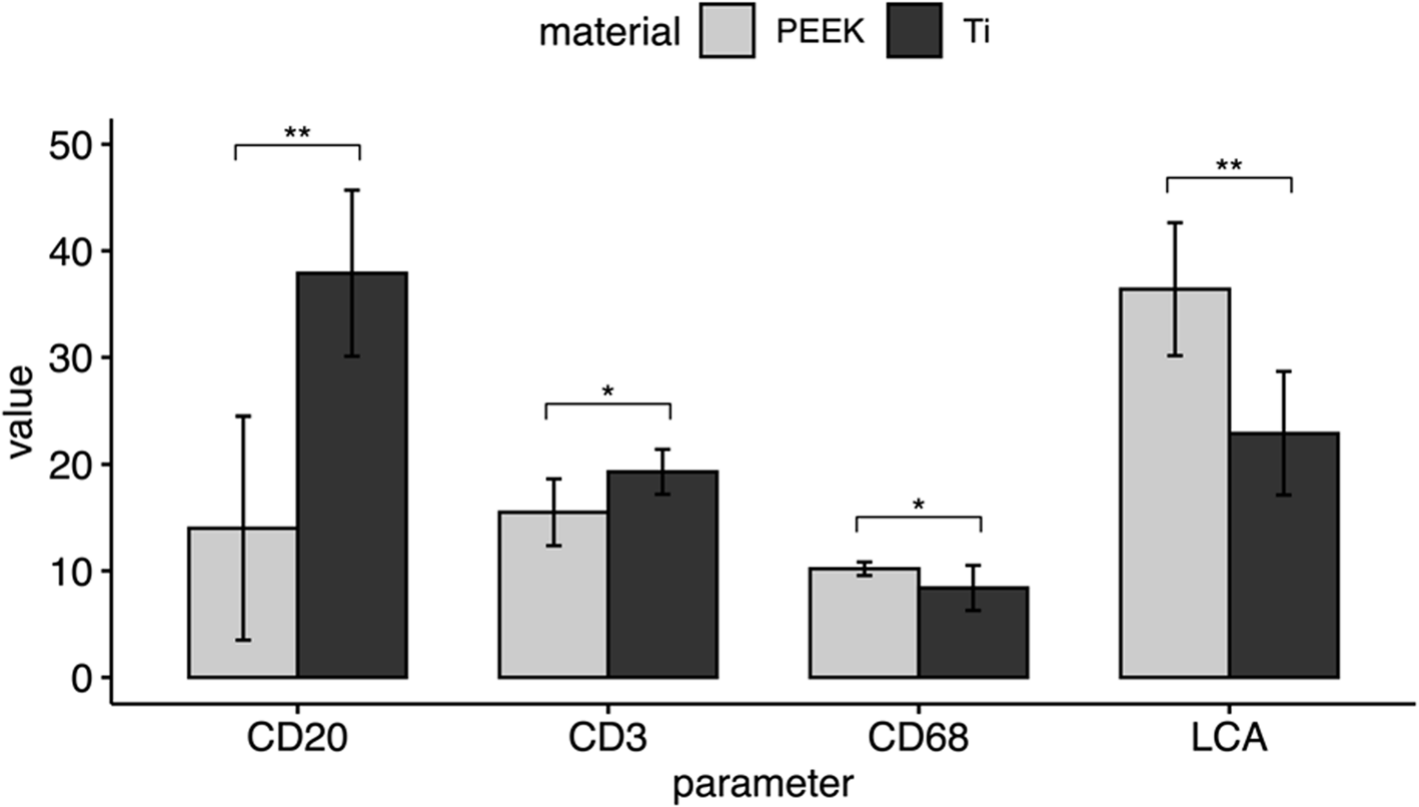
The number of LCA+, CD20+, CD3+ and CD68+ cells around PEEK and Ti healing abutments. Significant comparisons (*p* < 0.05) were emphasized on the graph

Immunohistochemical analyses showed mixed cellular infiltration consisting of lymphocytes (LCA+) and histiocytes (CD 68). The dominant was lymphocytic response resulting in 36.4 ± 6.24 LCA+/50 counted cells in group A and 22.9 ± 5.782 LCA+/50 counted cells in group B. A number of LCA+ and CD 68+ counted cells was significantly higher in group A in comparison to group B (The two-tailed paired Student’s t-test; *p* = 0.001 and *p* = 0.020, respectively). On the contrary, the number of CD 20+ and CD3+ counted cells was significantly greater in group B than in group A (Two-tailed Wilcoxon signed-rank test *p* = 0.006 and *p* = 0.010, respectively) (Table 2, Figs. 4 and 5).

**Table 2:** Mean and standard deviation for each continuous parameter, including the differences between PEEK and Ti

Parameter	Material	n	Mean	SD
CD20	PEEK	10	14	10.499
	Ti	10	37.9	7.795
	difference	10	−23.9	10.713
CD3	PEEK	10	15.5	3.136
	Ti	10	19.3	2.111
	difference	10	−3.8	4.211
CD68	PEEK	10	10.2	0.632
	Ti	10	8.4	2.119
	difference	10	1.8	2.044
LCA	PEEK	10	36.4	6.24
	Ti	10	22.9	5.782
	difference	10	13.5	9.312
BOP	PEEK	10	15.0	3.516
	Ti	10	10	5.273
	difference	10	5	5.829
PI	PEEK	10	12.5	4.396
	Ti	10	5.8	4.024
	difference	10	6.7	3.515

## Discussion

The results of the present study indicated the more pronounce soft tissue inflammatory reaction around PEEK HA, mirrored in an intensive sub-epithelium infiltrate around all PEEK HA. Additionally, LCA+ and CD 68+ cells dominated in PEEK group, while the number of CD 20+ and CD3+ cells was significantly higher around Ti HA.

Despite being a biologically inert material [28], earlier animal studies connected PEEK implantation to some inflammatory cells’ infiltration, occasionally with macrophages and foreign body giant cells, indicating mild chronical inflammatory response [10, 29]. Only few laboratory studies on animal model have investigated soft tissue response to PEEK surface, with somewhat opposing remarks. While one study concluded that the use of PEEK HA may be indicated [30], another demonstrated the significantly higher occurrence of multi-nucleated giant cells in contact with PEEK than with Ti closure caps [19]. However, to the best of authors’ knowledge, available literature data provide no information based on histological and immunohistochemical analyses of soft tissue interaction with PEEK in clinical settings.

Although soft tissue specimens were obtained from peri-implant soft tissue without clinically recognizable signs of inflammation, qualitative and semi-quantitative histological analyses demonstrated the presence of sub-epithelial inflammatory infiltrate around both PEEK and Ti HA, yet more pronounced around PEEK. Furthermore, leukocyte epithelial invasion was also observed in all specimens from PEEK group, less around Ti. Similar epithelial tropism in clinically healthy tissues has been previously reported in other studies [31, 32].

The sample examination in the present study showed the mixed inflammatory infiltrate consisted predominantly of lymphocytes, plasma cells and tissue histiocytes. The inflammatory response comparative analysis demonstrated the more intense inflammation display in the soft tissue surrounding PEEK, which reflected in significantly higher number of LCA+ and CD68+ cells around this material in comparison to Ti. It has been pointed out earlier that macrophages were the first cells coming in contact with implanted biomaterials, acting as important modulators of tissue biomaterial integration [33]. The observations of the present study might be in line with previous animal research that reported the heling abutment material influenced the multi-nucleated giant cells number, i.e. the PEEK closure caps surroundings had more of these cells than Ti [19]. It is important to emphasize that multi-nucleated giant cells are formed as a result of monocytes/macrophages fusion [34]. Furthermore, a recent immunohistochemical clinical study indicated that macrophages were considerably present in inflammatory infiltrate at peri-implantitis sites, suggesting their ability to increase peri-implant pocket depth following the tissue invasion, to disrupt the soft tissue adaptation and to enable bacterial invasion into the peri-implant tissue compartments [26, 35]. Therefore, the higher number of CD68+ cells observed in present study around PEEK closure caps might possibly be considered a risk factor for peri-implant pocket deepening, having in mind that a total bacterial load around PEEK was demonstrated to be higher than around Ti, although insignificantly [36]. Indeed, PEEK HA is present in the mouth only temporarily and relatively shortly to eventually participate in the establishment of peri-implantitis lesions. However, having in mind the above-mentioned ability of CD68+ cells to deepen the peri-implant pocket, their higher number around PEEK found in present study might be cautiously recognized as a potential contributor to peri-implant disease, although studies with a specified methodology are needed to confirm this assumption.

Additionally, further lymphocyte phenotyping in present research confirmed the significant dominance of CD 20+ B cells in inflammatory infiltrate around Ti, as well as the statistically greater number of CD 3 + T-lymphocytes in peri-implant soft tissue in contact to Ti compared to PEEK. However, as mentioned before, a total inflammatory infiltrate around Ti healing caps was less dense in comparison to PEEK, which mirrored in significantly higher number of LCA+ cells around PEEK material. These observations corroborate with findings of earlier studies where the detection of CD3+ T-lymphocytes in peri-implant soft tissue signified a well-controlled local immune response in otherwise clinically healthy tissue [31, 37]. The finding of humoral immunity domination around Ti HA in present study is comparable to previous research where also B lymphocytes were identified in greater number in contrast to T lymphocytes in peri-implant soft tissue clinically free of inflammation [32], even when comparing Ti and zirconia healing caps [38]. Additionally, the larger population of CD20+ cells might indicate that more lymphocytes were sensitized and matured to become plasma cells [23]. However, since CD20 cannot target the last stage of B cells development [32], further analyses are required to identify the presence of plasma cells.

Finally, it should be stressed out that despite the more pronounced inflammatory reaction was observed surrounding PEEK HA histologically, clinically soft tissues around both Ti and PEEK healing caps were free of inflammation at the moment of tissue specimens’ harvesting. Truly, BOP and PI values were significantly greater around PEEK HA at the time of biopsy, however with values up to 15%, clearly indicating the absence of clinically relevant soft tissue inflammation. Importantly, since this pilot study comprised a limited number of participants, only the further study conducted on a larger cohort of patients could confirm or contradict these results, including the observations made in present research on inflammatory cells’ profile detected. Furthermore, this study observed its subjects and placed implants during a three-month period, hence, no radiographic evaluation was performed. This limitation of the present study could be overcome in some future research with longer follow-up and radiographic assessment of a potential crestal bone changes.

## Conclusion

In conclusion, different HA materials generate different immunological response in the surrounding peri-implant soft tissues. The results of present study might lead to a conclusion that PEEK HA material encourages the more pronounced tissue inflammatory response demonstrated predominantly by histocytes’ and plasmacytes’ activation. On the other side, Ti triggers the inflammatory reaction of lower intensity, dominantly mediated by B-cells. The main limitation of the present study is the small sample size; therefore its findings should be interpreted with caution. The study continuation with greater sample size is needed to confirm the observations made in this pilot research.

## Data Availability

The datasets are available from the corresponding author on reasonable request.

## Abbreviations

PEEK: Polyetheretherketone
Ti: Titanium
MNGCs: Multinucleated giant cells
HA: Healing abutment
LCA: Leucocyte common antigen

## References

[1] Albrektsson T, Zarb G, Worthington P, Eriksson AR (1986). The long-term efficacy of currently used dental implants: a review and proposed criteria of success. Int J Oral Maxillofac Implants.

[2] Mombelli A, Muller N, Cionca N (2012). The epidemiology of peri-implantitis. Clin Oral Implants Res.

[3] Atieh MA, Alsabeeha NHM, Faggion CM, Duncan WJ (2013). The frequency of peri-implant diseases: a systematic review and meta-analysis. J Periodontol.

[4] Derks J, Håkansson J, Wennström JL, Klinge B, Berglundh T (2015). Patient-reported outcomes of dental implant therapy in a large randomly selected sample. Clin Oral Implants Res.

[5] Hahnel S, Wieser A, Lang R, Rosentritt M (2015). Biofilm formation on the surface of modern implant abutment materials. Clin Oral Implants Res.

[6] Berglundh T, Abrahamsson I, Welander M, Lang NP, Lindhe J (2007). Morphogenesis of the peri-implant mucosa: an experimental study in dogs. Clin Oral Implants Res.

[7] Welander M, Abrahamsson I, Berglundh T (2008). The mucosal barrier at implant abutments of different materials. Clin Oral Implants Res.

[8] Berglundh T, Lindhe J, Ericsson I, Marinello CP, Liljenberg B, Thomsen P (1991). The soft tissue barrier at implants and teeth. Clin Oral Implants Res.

[9] Abdallah MN, Badran Z, Ciobanu O, Hamdan N, Tamimi F. Strategies for optimizing the soft tissue seal around osseointegrated implants. Adv Healthc Mater. 2017;6(20):1700549. 10.1002/adhm.201700549 28960892

[10] Toth JM, Wang M, Estes BT, Scifert JL, Seim HB, Turner AS (2006). Polyetheretherketone as a biomaterial for spinal applications. Biomaterials..

[11] Skinner HB (1988). Composite technology for total hip arthroplasty. Clin Orthop Relat Res.

[12] Kern M, Lehmann F (2012). Influence of surface conditioning on bonding to polyetheretherketon (PEEK). Dent Mater.

[13] Rabiei A, Sandukas S (2013). Processing and evaluation of bioactive coatings on polymeric implants. J Biomed Mater Res A.

[14] Utzschneider S, Becker F, Grupp TM, Sievers B, Paulus A, Gottschalk O (2010). Inflammatory response against different carbon fiber-reinforced PEEK wear particles compared with UHMWPE in vivo. Acta Biomater.

[15] Najeeb S, Zafar MS, Khurshid Z, Siddiqui F (2016). Applications of polyetheretherketone (PEEK) in oral implantology and prosthodontics. J Prosthodont Res.

[16] Papathanasiou I, Kamposiora P, Papavasiliou G, Ferrari M (2020). The use of PEEK in digital prosthodontics: a narrative review. BMC Oral Health.

[17] Barkarmo S, Longhorn D, Leer K, Johansson CB, Stenport V, Franco-Tabares S (2019). Biofilm formation on polyetheretherketone and titanium surfaces. Clin Exp Dent Res.

[18] Barkarmo S, Östberg A-K, Johansson CB, Franco-Tabares S, Johansson PH, Dahlgren U (2018). Inflammatory cytokine release from human peripheral blood mononuclear cells exposed to polyetheretherketone and titanium-6 aluminum-4 vanadium in vitro. J Biomater Appl.

[19] Caballé-Serrano J, Chappuis V, Monje A, Buser D, Bosshardt DD (2019). Soft tissue response to dental implant closure caps made of either polyetheretherketone (PEEK) or titanium. Clin Oral Implants Res.

[20] van Brakel R, Meijer GJ, Verhoeven JW, Jansen J, de Putter C, Cune MS (2012). Soft tissue response to zirconia and titanium implant abutments: an in vivo within-subject comparison. J Clin Periodontol.

[21] Su-Mingxhsu O, Raine I, Fawger H (1981). Use of avidin biotin peroxidase complex (ABC) in immunoperoxidase technique: a comparison between ABC and unlabeled antibodies (PAP) procedures. J Histochem Cytochem.

[22] Gualini F, Berglundh T (2003). Immunohistochemical characteristics of inflammatory lesions at implants. J Clin Periodontol.

[23] Serichetaphongse P, Chengprapakorn W, Thongmeearkom S, Pimkhaokham A (2020). Immunohistochemical assessment of the peri-implant soft tissue around different abutment materials: A human study. Clin Implant Dent Relat Res.

[24] Calame KL (2001). Plasma cells: finding new light at the end of B cell development. Nat Immunol.

[25] Esposito M, Thomsen P, Mölne J, Gretzer C, Ericson LE, Lekholm U (1997). Immunohistochemistry of soft tissues surrounding late failures of Brånemark implants. Clin Oral Implants Res.

[26] Galarraga-Vinueza ME, Obreja K, Ramanauskaite A, Magini R, Begic A, Sader R (2021). Macrophage polarization in peri-implantitis lesions. Clin Oral Investig.

[27] Streuli M, Morimoto C, Schrieber M, Schlossman SF, Saito H (1988). Characterization of CD45 and CD45R monoclonal antibodies using transfected mouse cell lines that express individual human leukocyte common antigens. J Immunol.

[28] Wang H, Xu M, Zhang W, Kwok DTK, Jiang J, Wu Z (2010). Mechanical and biological characteristics of diamond-like carbon coated poly aryl-ether-ether-ketone. Biomaterials..

[29] Nieminen T, Kallela I, Wuolijoki E, Kainulainen H, Hiidenheimo I, Rantala I (2008). Amorphous and crystalline polyetheretherketone: Mechanical properties and tissue reactions during a 3-year follow-up. J Biomed Mater Res A.

[30] Rea M, Ricci S, Ghensi P, Lang NP, Botticelli D, Soldini C (2017). Marginal healing using Polyetheretherketone as healing abutments: an experimental study indogs. Clin Oral Implants Res.

[31] Tonetti MS, Imboden M, Gerber L, Lang NP (1995). Compartmentalization of inflammatory cell phenotypes in normal gingiva and peri-implant keratinized mucosa. J Clin Periodontol.

[32] Obădan F, Crăiţoiu Ş, Manolea HO, Hincu M-C, Iacov-Crăiţoiu MM (2018). The evaluation of the morphological evolution of the tissue integration of dental implants through conventional histology and immunohistochemistry techniques. Romanian J Morphol Embryol.

[33] Chehroudi B, Ghrebi S, Murakami H, Waterfield JD, Owen G, Brunette DM (2010). Bone formation on rough, but not polished, subcutaneously implanted Ti surfaces is preceded by macrophage accumulation. J J Biomed Mater Res A.

[34] Miron RJ, Bosshardt DD (2018). Multinucleated giant cells: good guys or bad guys?. Tissue Eng Part B Rev.

[35] Lang NP, Berglundh T (2011). Working Group 4 of the Seventh European Workshop on P. Periimplant diseases: where are we now?–Consensus of the Seventh European Workshop on Periodontology. J Clin Periodontol.

[36] Volpe S, Verrocchi D, Andersson P, Gottlow J, Sennerby L (2008). Comparison of early bacterial colonization of PEEK and titanium healing abutments using real-time PCR. Appl Osseoint Res.

[37] Seymour GJ, Gemmell E, Lenz LJ, Henry P, Bower R, Yamazaki K (1989). Immunohistologic analysis of the inflammatory infiltrates associated with osseointegrated implants. Int J Oral Maxillofac Implants.

[38] Bambini F, Santarelli A, Marzo G, Rubini C, Orsini G, Di Iorio D (2013). CD3 and CD20 expression in titanium vs zirconia peri-implant soft tissues: a human study. Eur J Inflamm.

